# Phase angle and body composition in long-term type 1 diabetes in adults: a comparative study in a Brazilian public reference outpatient clinic

**DOI:** 10.1186/s13098-024-01485-8

**Published:** 2024-11-13

**Authors:** Natália Fenner-Pena, Virgínia Capistrano Fajardo, Lívia Froes, Paulo Augusto Miranda Carvalho, Fábio Vasconcellos Comim, Viviane Sahade, Márcio Weissheimer Lauria, Henrique Oswaldo da Gama Torres

**Affiliations:** 1 Diabetes Academic League-Borges da Costa Ambulatory, Type 1 Diabetes Section or Department of Clinical Medicine, Federal University of Minas Gerais-UFMG, Belo Horizonte, Brazil; 2 Program in Sciences Applied to Adult Health, Federal University of Minas Gerais, Belo Horizonte, Brazil; 3 Endocrinologist at Clinic of Endocrinology and Metabology - Santa Casa, Belo Horizonte, Brazil; 4 Federal University of Bahia-UFBA, Salvador, Bahia Brazil

**Keywords:** Body composition, Bioimpedance, Phase angle, Type 1 diabetes

## Abstract

**Introduction:**

Type 1 Diabetes Mellitus (DM1) affects a small percentage of the population. Nevertheless, its prevalence is currently growing with alarming data on uncontrolled cases. The importance of body composition and Phase Angle (PA), assessed by Bioelectrical Impedance (BIA), in long- term DM1 patients lies in the fact that alterations in cellular integrity and body compartments may affect risk profiles and metabolic control. The objective of this study was to compare PA and body composition parameters between adults with DM1 and healthy controls.

**Methods:**

A comparative study was carried out in a public university outpatient clinic including a cohort of adult patients of both sexes diagnosed with DM1 and healthy controls matched by age and sex in a 2:1 ratio. Anthropometric measurements included weight, height and BMI. Using the raw BIA data of Resistance and Reactance, fat-free mass (FFM), fat mass (FM), fat-free mass index (FFMI), fat mass index (FMI), PA and standardized PA (SPA) were calculated. Means or medians were compared between the groups. Regression models were used to identify distinguishing characteristics of the groups and associations within the DM1 group (i.e. glycated hemoglobin (HbA1c), disease duration, presence of microvascular complications, capillary blood glucose, BMI and FMI).

**Results:**

88 patients with DM1and 46 healthy controls were evaluated. PA (6.05 vs. 6.85, *p* = 0.000) and SPA (-1.47 vs. -0,37, *p* = 0.000) were lower in patients with DM1 compared to healthy controls. People with DM1 displayed higher adiposity (%FM = 29.6 vs. 27.6, *p* = 0.016; FMI = 7.00 vs. 6.33, *p* = 0.016) and lower %FFM compared to healthy controls. Most of the differences were maintained after sex stratification; however, men with DM1 showed a lower FFMI than male controls (18.2 vs. 20.16, *p* = 0.029).

**Conclusion:**

Patients with DM1 present lower PA than healthy controls, which may be related to worse cell membrane integrity. Significant body composition differences between the groups and between sexes were identified, with data showing greater adiposity in women with DM1 and men displaying lower muscle mass. These findings suggest the importance of including PA and body composition evaluations in the follow-up of patients with DM1. The ultimate goal is to obtain a better metabolic control and, consequently, a better prognosis.

## Introduction

There is an increasing interest in the evaluation of body composition in adults with diabetes. Currently, there are only few studies evaluating distinct Bioelectrical Impedance (BIA) either direct or derived parameters in patients with long standing Type 1 Diabetes (DM1).

In different countries, evidence indicates that people with DM1, especially children and teenagers, have higher levels of adiposity, more intense weight gain and lower Phase Angle (PA) than healthy controls [1, 2, 5].

Phase angle is a direct BIA measurement that can indicate early alterations at cellular level and may thus become an important tool in the evaluation of a patient’s general health [6–8] and its association with diabetes’ complications and prognosis should be evaluated. BIA derived parameters, such as fat mass percentage and fat-free mass, may offer meaningful information about body composition and metabolism since alterations in body compartments may affect risk profiles and metabolic control [1–5]. This type of information can be acquired during routine outpatient clinical assessment and benefit the patient and the healthcare professional, as it can provide more reliable information with implications for prevention, clinical control and prognosis [8–12].

Since the DCCT study, weight gain and serum lipid levels in patients with diabetes have been related with inadequate eating practices, excess carbohydrates, lack of physical activity and exogenous insulin administration [5, 10].

Excess body weight can result from different sources including excessive adipose tissue and muscle hypertrophy; obese individuals with high body mass index (BMI) may present low muscle mass. Usually, healthcare professionals use only the BMI to evaluate obesity during outpatient anthropometric evaluation [13, 14]. However, BMI does not accurately measure adiposity nor presents a detailed evaluation of body composition, including fat mass and fat-free mass [17]. In patients with diabetes, such shortcomings are critical because increased fat deposition is associated with insulin resistance, while muscle mass plays an important role in general health and metabolic regulation. It is important to note that body composition in kilograms and percentages may not reflect specific changes in fat mass and fat-free mass. Parameters that take into consideration body size, such as fat mass and fat-free mass indexes (FMI and FFMI), might also have an important role in the patient’s general health and metabolic evaluation [17, 18, 23].

BIA derived FFMI has been used as criterion to define sarcopenia, and is strongly correlated with appendicular muscle mass measured by dual energy X-ray absorptiometry (DEXA). Although DEXA is considered the golden standard in assessing body composition, its applicability in outpatient-clinic settings, especially public services, is limited by its cost, radiation exposure and portability [18, 19]. Conversely, BIA is considered a safe and practical device to monitor body composition despite its limitations, such as the lack of precision to calculate body water in different conditions including diabetes. Based on the basic principle that body fluids and electrolytes conduct low tension electricity [12, 15, 18, 21, 22], BIA has additional advantages of being not only accessible and of low cost but also of being reliable and non invasive. Although some predictive BIA equations are not population specific, it may provide an assessment of body composition through the directly obtainable parameters of reactance and resistance.

According to the Brazilian Diabetes Society (SBD,2024), the treatment of DM1 is multidisciplinary. Each patient together with his/her family members and the healthcare team must be actively engaged in self-management and treatment planning to ultimately achieve a better body weight in addition to glycemic and general metabolic controls [35].

The most recent data from the International Diabetes Federations (IDF, 2023) indicate that 1.52 million people live with DM1 worldwide and their data on adult patients present an evident research gap [36]. Moreover, according to the IDF the life expectancy of a person with DM1 in countries like Brazil may be about 15 years lower than that of people with DM1 in developed countries, indicating the need for better health care initiatives and treatment in developing countries.

DM1 is a chronic autoimmune disease and its management has been associated with significant changes in body composition. The main objective of this study is to investigate these body composition changes in a cohort of patients with long standing DM1 seen at a public-university outpatient referral clinic, in an attempt to contribute to the understanding of their role in the health of this specific group of patients.

BIA direct (i.e. PA, standardized PA, SPA) and indirect parameters (i.e. percentage of fat-free mass, percentage of fat mass, FFMI and FMI) were compared in long standing DM1 outpatients and healthy controls. Knowledge of differences in body composition of patients with DM1 may be of critical importance since they may be related to the patient’s health and organ integrity in the case of PA and SPA, or to metabolic control and prognosis in the case of fat-free mass and fat mass parameters. There is a lack of data on this subject in the adult DM1 population and, specifically, in the Brazilian population.

## Methods

This is a comparative study carried out in a public-university outpatient referral clinic, which included a cohort of adult patients of both sexes diagnosed with DM1 and a control group of healthy individuals matched by age and sex on a 2:1 ratio. Data were collected from March 2020 to March 2023. Patients were selected from the Diabetes League, a trained multidisciplinary team composed of students, faculty and university personnel from the areas of medicine, nutrition, and pharmacy that develops and delivers to diabetic patients orientation activities in nutrition, foot care, physical activity, and insulin use. Patients or their proxies and controls signed the Informed Consent Form (ICF) previously approved by the Institutional Ethics Research Committee. Exclusion criteria included pregnancy, age under 18 years, hypersensitivity to electronic devices, bedridden patients or with a disability that made it impossible to properly place BIA electrodes, patients with chronic kidney disease (CKD) on dialysis therapy, and patients with cardiovascular disease in use of a pacemaker. Moreover, patients with anemia, edema, heart failure, nephrotic syndrome, and cirrhosis were also excluded. The healthy control group was recruited among medical students and university personnel. Patients with DM1 were assessed as part of a nutritional appointment, which included glycated hemoglobin (HbA1c) result, BIA analysis, capillary blood glucose, body weight, height, and body mass index. In addition, a structured questionnaire was administered and included questions regarding the patient’s health background, time of DM1 diagnosis, food intake record, average 24-hour consumption of carbohydrates, and presence of microvascular complications (the presence or any association of retinopathy, neuropathy or non-nephrotic nephropathy). Individuals in the healthy control group received anthropometric and BIA evaluations.

### Nutritional status assessment

Body weight and height were obtained with a Tanita® scale by means of adequate and standardized procedure. BMI was calculated by the standard WHO formula of weight/height^2^ (Who Expert Committee on Physical Status 1995).

## Bioelectrical impedance (BIA)

Low intensity (800 µA), single frequency (50 kHz) BIA analysis was performed (Quantum X device, RJL systems, 2007). Resistance (R), reactance (Xc), and PA values were obtained by the Body Composition software as proposed by the device manufacturer. A standard procedure was employed, including position of body and members, removal of metals in contact with skin, cleansing with alcohol, and location of electrodes (Kyle 2004; WHO Expert Committee on Physical Status 1995). SPA was calculated according to the following Eq. (5):

$$\:\text{S}\text{P}\text{A}=\frac{\left[\text{P}\text{A}-\text{m}\text{e}\text{a}\text{n}\:\text{P}\text{A}\:\left(\text{f}\text{o}\text{r}\:\text{a}\text{g}\text{e}\:\text{a}\text{n}\text{d}\:\text{g}\text{e}\text{n}\text{d}\text{e}\text{r}\right)\right]}{\text{p}\text{o}\text{p}\text{u}\text{l}\text{a}\text{t}\text{i}\text{o}\text{n}\:\text{s}\text{t}\text{a}\text{n}\text{d}\text{a}\text{r}\text{d}\:\text{d}\text{e}\text{v}\text{i}\text{a}\text{t}\text{i}\text{o}\text{n}\:\left(\text{f}\text{o}\text{r}\:\text{a}\text{g}\text{e}\:\text{a}\text{n}\text{d}\:\text{g}\text{e}\text{n}\text{d}\text{e}\text{r}\right)}$$

FFM was obtained through the Body Composition Program of the BIA device, according to the following formula: [17, 25]

$$\begin{aligned}\text{F}\text{F}\text{M}&=-4.104+\left(\frac{{0.518\times\text{h}\text{e}\text{i}\text{g}\text{h}\text{t}}^{2}}{\text{r}\text{e}\text{s}\text{i}\text{s}\text{t}\text{a}\text{n}\text{c}\text{e}}\right)\\ &\quad+\left(0.231\times\text{w}\text{e}\text{i}\text{g}\text{h}\text{t}\right)+\left(0.130\times\text{r}\text{e}\text{a}\text{c}\text{t}\text{a}\text{n}\text{c}\text{e}\right)\\ &\quad+\left(4.229\times\text{s}\text{e}\text{x}\right);\text{m}\text{e}\text{n}=1,\:\text{w}\text{o}\text{m}\text{e}\text{n}=0;\end{aligned}$$

$$\:\text{F}\text{F}\text{M}\:\text{p}\text{e}\text{r}\text{c}\text{e}\text{n}\text{t}\text{a}\text{g}\text{e}\:\left(\text{\%}\text{F}\text{F}\text{M}\right)=\frac{\text{F}\text{F}\text{M}\:\left(\text{k}\text{g}\right)}{\text{w}\text{e}\text{i}\text{g}\text{h}\text{t}\:\left(\text{k}\text{g}\right)}\times100$$

$$\:\text{F}\text{F}\text{M}\text{I}=\frac{\text{F}\text{F}\text{M}}{{\text{h}\text{e}\text{i}\text{g}\text{h}\text{t}}^{2}}$$

Plasma HbA1C concentrations were measured by a method certified by the National Glycohemoglobin Standardization Program (NGSP).

### Statistical analysis

Data were presented as absolute values and frequencies, mean *±* SD (standard deviation) or median, and percentiles depending on the distribution according to Shapiro-Wilk test.

Logistic regressions were carried out having the DM1 and control groups as dichotomous outcomes to verify, within the studied sample, which factors were clearly associated with one of the outcomes. Receiver operating characteristic (ROC) curves were also performed to evaluate the behavior of PA and SPA in distinguishing between patients with diabetes and healthy controls and to investigate possible cutoffs associated with a higher probability of belonging to the DM1 group.

Linear regression was used in the DM1 group to evaluate the association between the independent variables (HbA1c, duration of disease, presence of microvascular complications, capillary blood glycemia on the day of BIA application, BMI and FFMI) and PA [21].

Due to their strong collinearity, different models, both of logistic and linear regression, were performed separately for each body composition parameter (BMI, FFM and FM percentages, FFMI and FMI).

All analyses used the package SPSS®19.0 (Chicago: SPSS Inc. IBM Corp). A significance level of 5% was considered. Sample size calculation (G*Power 3.1.9.4) assumed an effect size of 0.47 for the difference of PA means between the DM1 and control groups [5]. A power of 80% and an alpha error of 0.05, resulted in a sample size of 43 controls and 85 cases.

## Results

**Fig. 1:**
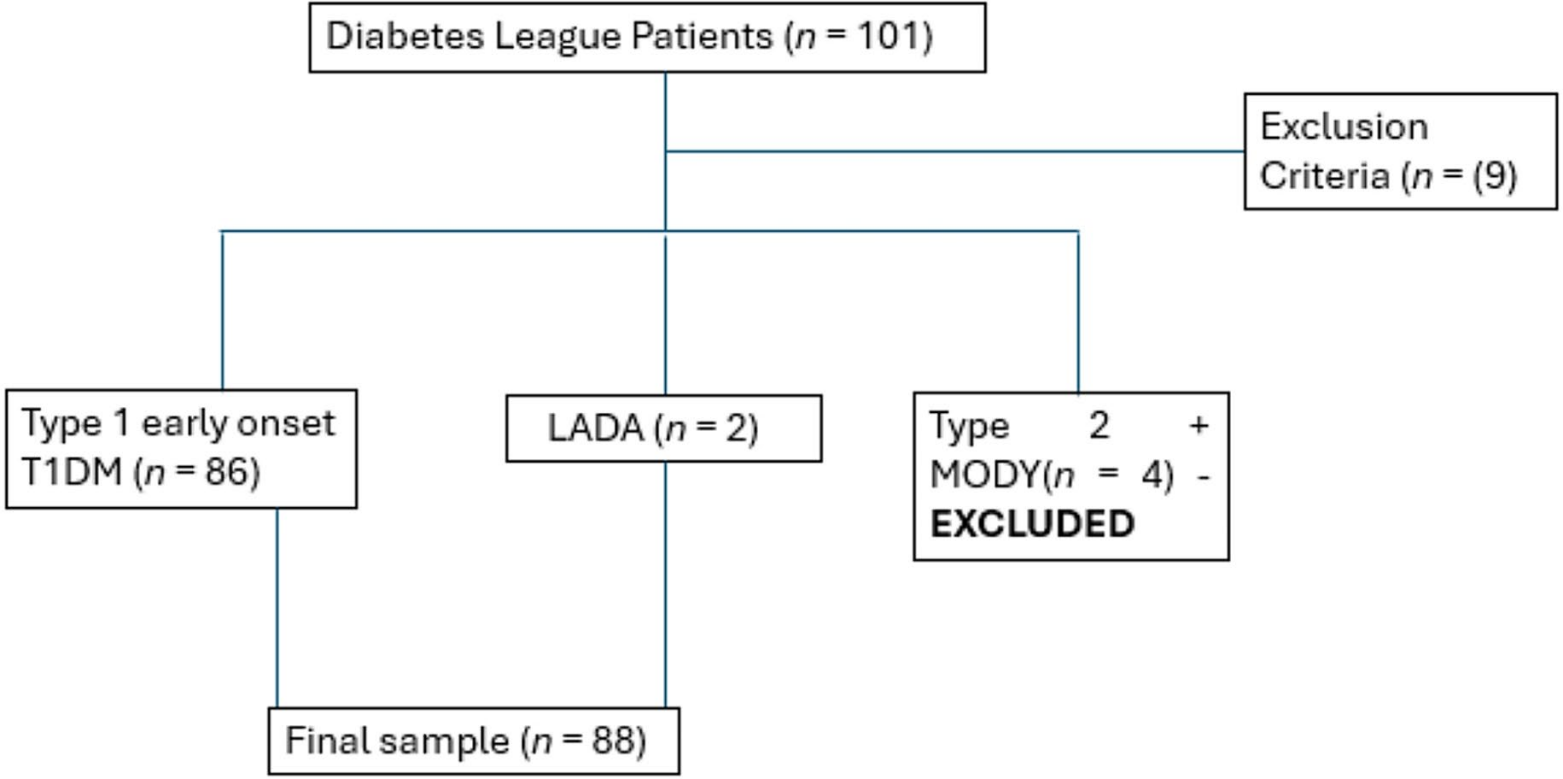
Diagram for selection sample

The final sample was achieved according to the diagram on Fig. 1.

The cohort of 88 patients with long-term DM1 (21.2 *±* 9.3 years) was compared with a group of 46 controls, matched by sex and age (Table 1). Most patients with diabetes were uncontrolled according to HbA1c levels (8.96 *±* 2.09%), and 44.5% had microvascular complications.

**Table 1:** Demographic and clinical data

	DM1 (*n* = 88)			C (*n* = 46)		*p*
Age*	36.2 *±* 11.3			36.0 *±* 11.7		0.914
Duration* (years, min- max)	21.2 ± 9.3 (5–48)			-		-
HbA1C* (min- max)	8.57 *±* 1.84(5.50–15.50)			-		-
Complications**	40 (44.5%)			-		-
	**F**	**M**	***p***	**F**	**M**	***p***
Sex**	47 (53.4%)	41 (46.6%)	-	29 (63.0%)	17 (37.0%)	0.285
Age (years)	33.1 *±* 8.0	39.7 *±* 13.3	**0.006**	34.0 *±* 9.2	39.2 *±* 14.9	0.205
Duration of diabetes (years)	20.7 ± 9.6	21.8 ± 9.0	0.574	-	-	-
HbA1C*	8.96 *±* 2,09	8.12 *±* 1.40	**0.032**	-	-	-
Complications**	19 (40.4%)	21 (51.2%)	0.310	-		-

HbA1C (Glycated Hemoglobin); DM1 (Diabetes Type 1 Group); C (Control Group)

* = Student’s T test; ** = Chi square

**Fig. 2:**
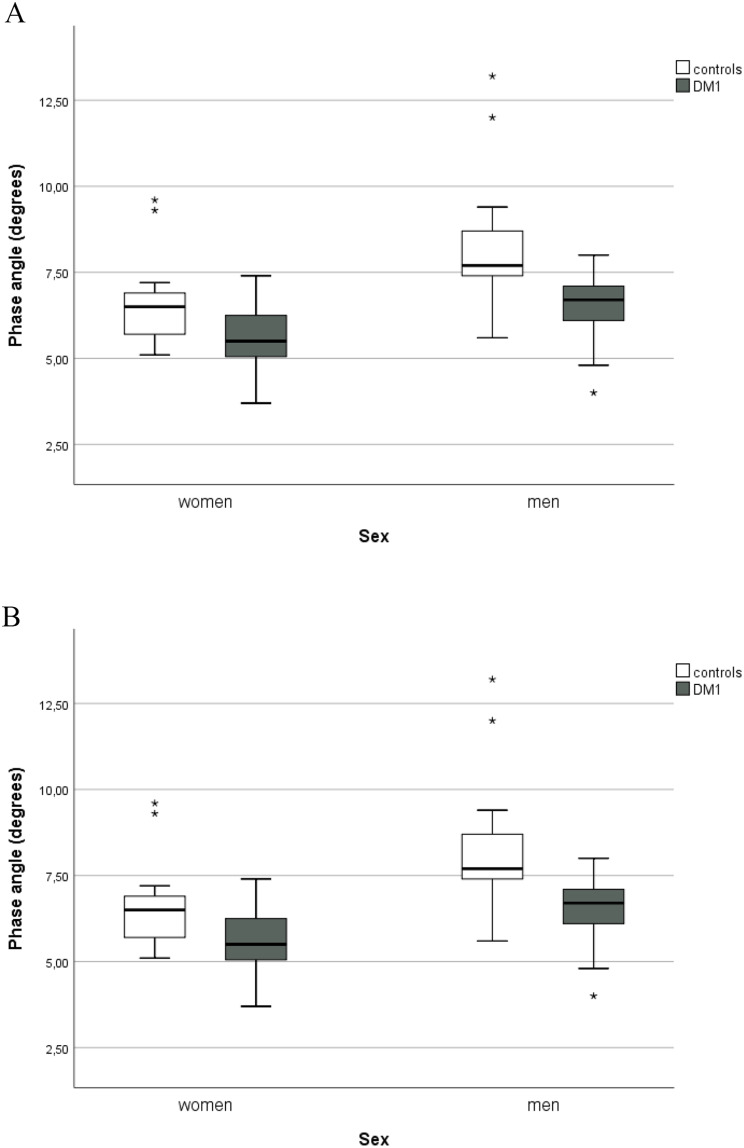
Box plot of PA and SPA in DM1 and control groups. **A** PA distribution according to groups (DM1 and healthy controls) and sex; **B** SPA distribution according to groups (DM1 and healthy controls) and sex

BIA parameters showed significantly higher PA and SPA medians in the control group (Table 2; Fig. 2), and significant differences in body composition were observed. In patients with DM1, fat-free mass was significantly lower according to %FFM, and body fat significantly higher, according to %FM and FMI (Table 2).

When groups are stratified by sex, differences in PA and SPA remained significant, and differences in body composition seemed to be mainly due to a lower %FFM and a higher %FM observed in women with DM1. When FMI was evaluated, a higher fat content in women with DM1, but not in men, was confirmed. A lower FFM content, measured by FFMI, was shown in men with DM1; however, the same was not observed in women (Table 2).

**Table 2:** BIA parameters in patients with diabetes and healthy control

	DM1			Controls		p**
BMI*	23.39 (21.98; 27.19)			23.44 (20.89; 24.68)		0.174
PA*	6.05 (5.20; 6.80)			6.85 (6.00;7.63)		**< 0.001**
SPA*	-1.465 (-2.129; − 0.778)			− 0.365 (-1.072.137)		**< 0.001**
%FFM*	70.40 (62.93;75.20)			72.40 (67.95;81.08)		**0.016**
%FM*	29.60 (24.80;37.08)			27.60 (18.93;32)		**0.016**
FFMI*	16.62 (15.17;18.58)			16.69 (15.32;19.00)		0.653
FMI*	7.00 (5.53;9.40)			6.33 (4.09;7.57)		**0.016**
	**F**			**M**		
	**DM1(47)**	**C (29)**	p**	**DM1(41)**	**C (17)**	p**
BMI*	23.20 (21.58;30.28)	22.52 (19.82;23.59)	**0.014**	23.49 (22.14;26.81)	24.86 (23.97;27.82)	0.140
PA*	5.50 (5.00;6.30)	6.50 (5.70;6.95)	**< 0.001**	6.70 (6.05;7.10)	7.70 (7.05;8.90)	**< 0.001**
SPA*	-1.624 (-2.226;-0.739)	-0.482 (-1.079;0.036)	**< 0.001**	-1.424 (-2.100;-0.779)	-0.027 (-0.847;1.095)	**< 0.001**
%FFM*	65.10 (59.30;69.80)	71.20 (67.75;78.10)	**< 0.001**	74.30 (70.65;76.9)	74.60 (67.90;86.45)	0.505
%FM*	34.90 (30.20;40.70)	28.8 (21.90;32.25)	**< 0.001**	25.70 (23.10;29.35)	25.40(13.55;32)	0.505
FFMI*	15.41 (14.90;1731)	16.08 (14.73;16.74)	0.987	18.02 (16.50;19.85)	20.16 (16.83;21.86)	**0.029**
FMI*	8.21 (6.65;11.54)	6.30 (4.35;7.49)	**0.001**	6.13 (5.36;7.21)	6.36 (3.95;8.04)	0.713

F = female; M = male; BMI = body mass index; PA = phase angle; SPA = standardized phase angle; %FFM = percentage of fat-free mass; %FM = percentage of fat mass; FFMI = fat-free mass index; FMI = fat mass index; * = median (p25;p75); ** = Mann Whitney test

Effect sizes were calculated for PA and SPA according to sex and the entire sample. Considering the non-normal distribution of the samples and the use of the nonparametric Mann Whitney test to compare medians [37], the following results were obtained: PA (women) = 0.47 (CI: 0.24–0.69); PA (men) = 0.49 (CI: 0.23–0.74); SPA (women) = 0.49 (CI: 0.26–0.71); SPA (men) = 0.50 (CI: 0.24–0.76); PA (entire sample) = 0.36 (0.19–0.53); SPA (entire sample) = 0.50 (0.11–0.89). These effect sizes can be considered medium to large when calculated by sex [37]. Confidence intervals were larger when the entire sample was analyzed, probably due to the larger variance when sexes are combined.

Results of PA and SPA between groups are illustrated in Fig. 2.

Logistic regression was carried out to assess the effect of PA or SPA on the likelihood of belonging to DM1 or control group (Table 3). The models were controlled for age and sex, and different parameters of body composition (i.e. %FFM, %FM, FFMI and FMI) were entered in separate models, along with PA (or SPA), age and sex. Both PA and SPA were associated with a high discriminatory capacity indicating an 80% decrease in the chances of being part of the DM1 group for every 1 unit increase in PA and a 75% decrease in the chance of being part of the DM1 group for every 1 unit increase in SPA.

**Table 3:** Discriminatory capacity of PA or SPA in distinguishing DM1 and control subjects: logistic regression models according to the body composition parameter entered

	Body composition parameter	Variables in the final model	Exp(B)	*p*
Phase angle	%FFM*	PA, %FFM, sex	0.234 (0.127-0.433)	< 0.001
	FFMI	PA, sex	0.201(0.105-0.388)	< 0.001
	FMI	PA, FMI, sex	0.197 (0.102-0.379)	< 0.001
Standardized phase angle	%FFM*	SPA, %FFM, sex,	0.283 (0.167-0.480)	< 0.001
	FFMI	SPA, FFMI	0.248(0.144-0.427)	< 0.001
	FMI	SPA, FMI	0.244(0.139-0.429)	< 0.001

PA = phase angle; SPA = Standardized phase angle; %FFM = fat free mass percentage; FFMI = fat free mass index

FMI = fat mass index

* % fat free mass percentage is the inverse of fat mass percentage (%FFM = 100 - %FM), therefore results are equal for both parameters and were not shown for %FM

The overall models were statistically significant when compared to the null model and explained 38–46% of the group allocation (DM1 or control subjects) for PA and 39–46% for SPA. The models correctly predicted about 80% of cases in both PA and SPA models.

ROC curves for both PA (AUC = 0.719, 0.629–0.808, *p* = 0.000), and especially for SPA (AUC = 0.805, 0.729–0.881, *p* = 0.000), also illustrate the mentioned discriminatory capacity. Among control individuals, only 9 (19.6%) had SPA values below the cutoff of -1.145 obtained with the Youden method, while 58 (65.9%) DM1 patients displayed SPA below that threshold. Only 3 (6.5%) healthy controls showed SPA below the threshold of -1.65, which corresponds to the 5th percentile of Brazilian population, while 41(46.6%) DM1 patients had results below this cutoff [38].

Linear regression models were used in the DM1 group to analyze associations between clinical and anthropometric data (i.e. sex, age, duration of disease, rate of microvascular complications, anthropometric variables, HbA1c and capillary blood glycemia) and PA or SPA. No statistically significant association was demonstrated between HbA1C levels and PA or SPA.

## Discussion

This study aimed to evaluate PA and body composition in adult DM1 patients with long-term disease in comparison to healthy individuals, matched according to age and sex.

It is known that DM1 is associated with an elevated risk for complications, and that poor metabolic control determined by HbA1c is correlated differently with acute and chronic complications. Body composition and PA have not been thoroughly evaluated in adult DM1 patients with long standing disease.

PA is calculated based on the direct BIA measurements of R and Xc. PA data in patients with DM1 are scarce and have not been studied in a Brazilian population.

The study results showed lower PA values in patients with DM1 compared to healthy controls, similar to younger patients with DM1, as found by N’Samba et al. in children and adolescents with recently diagnosed DM1. Buscemi et al. [12], studying patients with both DM1 and DM2 found significantly lower PA values in young male patients with DM1, but not in females. In the present study, when PA values were adjusted for age and sex, by either logistic regression or the use of SPA, the difference between patients with DM1 and controls in both sexes remained significant. A thorough search in different databases (PubMed, Scopus, EMBASE) for studies using SPA showed that it had not been used in diabetes, and the present results indicate its potential usefulness. Among control patients, 6.5% showed values below the SPA threshold of -1.65, which corresponds to the 5th percentile of the Brazilian population, in comparison to 46.6% of patients with DM1.

Besides confirming lower PA values in patients with DM1 in general, the study is in agreement with others that show lower values in women with diabetes (Dittmar et al. [19]. This is probably related to the body composition, with a lower amount of FFM.

Ditmar et al. [19] found an inverse relationship between PA and HbA1c that was attributed to catabolic state and poor control. Contrary to our initial hypothesis, an association between PA and HbA1c could not be demonstrated, possibly because the study was not powered enough for linear regression, due to the number of independent variables. Furthermore, the sample was mainly composed of uncontrolled patients, which may have prevented conclusions that could have been obtained with the presence of better controlled ones.

Important differences in body composition were observed between patients with DM1 and healthy controls. DM1 patients had an excess of fat mass, in terms of %FM and FMI, mainly due to the female component of the sample. Body fat increases substantially in females during puberty, and may be especially marked in patients with diabetes [33]. This finding may be related to multiple-dose insulin regimens, carbohydrate-rich diets and possibly to the inflammatory activity and insulin resistance/metabolic syndrome, which result in low muscle mass, increased fat mass and poor diabetes control evaluated by HbA1c [33, 34].

In this study, a lower FFMI in men with DM1was shown compared to healthy male controls. A high prevalence of low muscle mass evaluated by appendicular lean mass index observed in a cohort of long standing DM1 patients, similar to that found in the older age group of the general population (but with 25 years in advance), suggests a possible pathogenetic role of DM1 on muscle trophism and function [39, 40]. If FFMI should be considered a surrogate marker of muscle mass as indicated [16], the finding of a lower FFMI in DM1 patients may be of clinical importance and should be further evaluated.

These alterations in body composition may determine a future impact on overall health with various metabolic derangements, such as dyslipidemia, arterial hypertension, sarcopenia and insulin resistance. Muscle mass reduction results in a significant impact on insulin sensitivity, glucose and lipid processing and basal metabolic rate, with consequences on metabolic stability in DM1.

A recent study [31] carried out in patients with DM1 highlighted the need for attention to women’s metabolic care and body composition, since female patients showed higher cardiovascular risk than male patients, contrary to usual expectations [32]. The discrepancy observed in the study was explained by the high prevalence of chronic complications in the sample, mainly diabetic retinopathy, a factor that categorizes a patient as having high cardiovascular risk (SBEM - Brazilian Society of Endocrinology and Metabology). Furthermore, women with diabetes had a higher prevalence of kidney disease, as well as worse glycemic control and slightly higher levels of LDL cholesterol, which could justify a higher proportion of female patients classified as high cardiovascular risk when evaluated by the Steno T1 Risk Engine (ST1RE), used to predict cardiovascular events in patients with DM1 [31]. Whether these observations could be explained by body composition alterations that may be more profound in women than in men remain to be further clarified. That said, this seems to be an important line of investigation, with some evidence pointing towards this direction [24].

This is a challenging topic, where definite conclusions cannot be made at this time. Additional studies developing body composition evaluation protocols may contribute to a better metabolic control in patients with DM1.

## Conclusion

In the present study, patients with long-term DM1 presented with lower PA compared to a cohort of healthy individuals. In terms of body compartment percentages, patients with DM1 showed higher FM and lower FFM. When corrected by body size, patients with DM1 showed higher FM compared to healthy individuals. Significant body composition differences between sexes were noted, with data showing greater adiposity in women with long-term DM1 in relation to healthy females, and men showing lower FFM corrected by height compared to healthy males.

These findings indicate the importance of careful and regular body composition evaluations in patients with DM1. The ultimate goal is to contribute to a better metabolic control and prognostic in patients with long-term DM1, considering the data associating disease duration with PA, which is a marker of general health. Multidisciplinary outpatient follow-ups should benefit from this approach.

## Data Availability

No datasets were generated or analysed during the current study.
